# Characterization of novel extracellular proteases produced by *Acanthamoeba castellanii* after contact with human corneal epithelial cells and their relevance to pathogenesis

**DOI:** 10.1186/s13071-024-06304-7

**Published:** 2024-05-29

**Authors:** Alvie Loufouma-Mbouaka, Tania Martín-Pérez, Martina Köhsler, Zeynep Danisman, Maya Schwarz, Rounik Mazumdar, Ascel Samba-Louaka, Julia Walochnik

**Affiliations:** 1 Center for Pathophysiology, Infectiology and Immunology, Institute of Specific Prophylaxis and Tropical Medicine, Medical University of Vienna, Vienna, Austria; 2 Max Perutz Labs Vienna, Department of Medical Biochemistry, Medical University of Vienna, Vienna, Austria; 3 Laboratoire Ecologie Et Biologie Des Interactions, Université de Poitiers, UMR CNRS, 7267, Poitiers, France; 4 Present Address: GenomeByte Ltd, London, UK

**Keywords:** *Acanthamoeba*, Human corneal epithelial cells, Host‐pathogen interaction, Serine protease, Metalloproteinase, Protease inhibitor, Virulence factors, Pathogenesis, Mannose-binding protein

## Abstract

**Background:**

Proteases produced by *Acanthamoeba* spp. play an important role in their virulence and may be the key to understanding *Acanthamoeba* pathogenesis; thus, increasing attention has been directed towards these proteins. The present study aimed to investigate the lytic factors produced by *Acanthamoeba castellanii* during the first hours of in vitro co-culture with human corneal epithelial cells (HCECs).

**Methods:**

We used one old and one recent *Acanthamoeba* isolate, both from patients with severe keratitis, and subsets of these strains with enhanced pathogenic potential induced by sequential passaging over HCEC monolayers. The proteolytic profiles of all strains and substrains were examined using 1D in-gel zymography.

**Results:**

We observed the activity of additional proteases (ranging from 33 to 50 kDa) during the early interaction phase between amoebae and HCECs, which were only expressed for a short time. Based on their susceptibilities to protease inhibitors, these proteases were characterized as serine proteases. Protease activities showed a sharp decline after 4 h of co-incubation. Interestingly, the expression of *Acanthamoeba* mannose-binding protein did not differ between amoebae in monoculture and those in co-culture. Moreover, we observed the activation of matrix metalloproteinases in HCECs after contact with *Acanthamoeba*.

**Conclusions:**

This study revealed the involvement of two novel serine proteases in *Acanthamoeba* pathogenesis and suggests a pivotal role of serine proteases during *Acanthamoeba*-host cell interaction, contributing to cell adhesion and lysis.

**Graphical Abstract:**

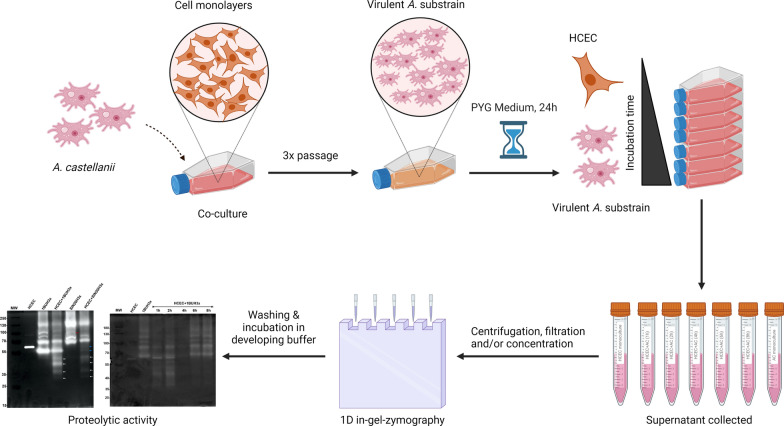

**Supplementary Information:**

The online version contains supplementary material available at 10.1186/s13071-024-06304-7.

## Background

*Acanthamoeba* keratitis (AK) is a serious ocular infection caused by pathogenic free-living amoebae belonging to the genus *Acanthamoeba*, which occur naturally in diverse habitats worldwide, including aquatic environments, soil, sand, dust, and even the air. Most cases of AK are associated with contact lens use; these amoebae may contaminate contact lenses and invade and infect the human cornea, in some cases leading to a total loss of vision [1, 2]. Nevertheless, AK is also reported in non-contact lens wearers. The frequency of occurrence of AK has increased worldwide in many countries during the past decades [3–6], and this infection still represents a challenge for healthcare professionals. The current therapeutic regimen involves the use of disinfectants and antiseptics, e.g. a combination of biguanides with diamidines, and is lengthy and often not entirely effective [2, 7, 8]. Treatment failure and persistence of infection are primarily due to cyst formation and the fact that the amoebae can migrate deeply into the corneal stroma. However, little is known about how these pathogens cross the corneal epithelium without the need for prior corneal abrasion and how they perforate the Bowman membrane [2, 9–13].

Various in vitro and in vivo studies suggest that the first step in the pathogenesis of AK is adhesion to the corneal epithelial cells. Adhesion is assumed to be mediated by the so-called mannose-binding protein (MBP)—a 130-kDa molecule expressed on the surface of the amoeba—and other adhesins, and to lead to the release of extracellular proteases, especially serine proteases and metalloproteases [10, 14, 15]. These proteases are of special interest because of their key role in the survival and virulence of *Acanthamoeba* in the human eye, including invasion and penetration of host tissue, degradation of proteins and modulation of the host immune response [2, 9]. However, *Acanthamoeba*’s cytopathic potential is not constant; it may decline over time after prolonged axenic culture in the laboratory and be reactivated by contact with mammalian cells [16, 17].

Despite significant research progress in recent years, the mechanisms by which the amoebae lose their pathogenic potential or restore their attenuated virulence to be able to invade host tissue and induce cytolysis are still not well understood. The mechanism is believed to be associated with epigenetic modifications such as histone modification, DNA methylation, and non-coding RNA-associated gene silencing, which lead to an increase or decrease in gene expression [18, 19]. *Acanthamoeba* produces various proteases of different types, but little is known about their role in the pathological mechanisms [20–22].

To better understand the interactions between *Acanthamoeba* and human cells and to systematically assess the role of secreted proteases during the early phase of their interaction, we used 1D in-gel zymography to explore the proteolytic profiles of pathogenic *Acanthamoeba* strains in contact with human corneal epithelial cells (HCECs). Moreover, we determined whether the additional lytic factors observed during the first hours of co-culture with HCECs correlated with the expression of *MBP*.

## Methods

### Chemicals

Phenylmethylsulfonyl fluoride (PMSF), E64 (N-[N-(L-3-transcarboxyirane-2-carbonyl)-L-Leucyl]-agmatine), phenanthroline, tosyl-lysine chloromethyl ketone hydrochloride (TLCK) and other chemical compounds were purchased from Sigma (Sigma-Aldrich Handels GmbH, Vienna, Austria) unless otherwise stated. CellTiter 96^®^ AQueous One Solution Reagent was purchased from Promega (Promega, Madison, WI, USA).

### Media and cell line

Media and components were purchased from Innoprot (Derio, Bizkaia, Spain). Immortalized HCECs (P10871-IM) were obtained from the Department of Biomedicine and Biotechnology, Faculty of Pharmacy, University of Alcalá, Madrid, Spain (courtesy of Professor Jorge Pérez-Serrano). Cells were subcultured in CEpiCM (P60189) containing 5% foetal bovine serum, 1% epithelial cell growth supplement and 1% penicillin/streptomycin, maintained at 37 °C and 5% CO2. T25 and T75 flasks were coated with a thin layer of type I collagen (P8188) to enhance cell attachment and proliferation.

### *Acanthamoeba* strains and culture conditions

The clinical *A. castellanii* strains 1BU (ATCC PRA-105) and SIN20, both genotype T4, were isolated from two different patients diagnosed with infectious keratitis in 1998 and 2020, respectively. Both strains were maintained and grown axenically in proteose peptone–yeast extract–glucose (PYG) medium, containing 10 g proteose peptone, 10 g yeast extract, 5 g NaCl, 5 g glucose, 0.7 g Na_2_HPO_4_ and 0.7 g KH_2_PO_4_ per litre, and maintained at 34 °C.

### Reactivation of *Acanthamoeba* virulence by passage over human corneal epithelial cell monolayer

Immortalized HCECs (1 × 10^6^ cells) were cultivated and grown in a 75-cm^2^ tissue culture flask until the formation of a tissue monolayer. Before starting the experiment, the HCEC culture medium was replaced by serum-free CEpiCM. Next, 2.5 × 10^5^ *Acanthamoeba* trophozoites (resuspended in serum-free CEpiCM) were added to the monolayer and incubated at 34 °C with 5% CO_2_ until the cell monolayer was completely lysed. This procedure was performed three times sequentially, and the resultant substrains 1BUH3x and SIN20H3x were considered virulent (cytopathic) and maintained in PYG medium for 24 h before the subsequent experiment.

### Human corneal epithelial cell-*Acanthamoeba* co-culture and protease extraction

Before the assay, HCECs (5 × 10^5^) were inoculated into a T25 flask for cell culture and incubated overnight. The following day, the culture medium was replaced with a serum-free culture medium after washing with Dulbecco’s phosphate-buffered saline (DPBS; 1 ×). Next, 1 × 10^6^ trophozoites per strain from the virulent substrains (1BUH3x and SIN20H3x) were added to HCECs and incubated for various periods (1 h, 2 h, 4 h, 6 h and 8 h). Monocultures containing either trophozoites alone or HCECs alone were used as controls.

At each time point, the culture medium was collected, transferred to a sterile 15-ml Falcon tube and centrifuged at 700 × *g* to remove the amoebae and HCECs. The supernatant was filtered through a 0.22-µM cellulose acetate filter (Membrane Solutions, USA) to obtain a cell-free filtrate containing excreted proteases and concentrated (except for supernatants collected after 4 h, 6 h and 8 h of co-culture, which contained high protein concentration) from an initial volume of 6 ml to 200 µl using a Vivaspin 6 centrifugal concentrator (3000 MWCO, Sartorius Stedim Lab Ltd., UK). The final volume was collected, and 2.5–5 µg of protein was used for subsequent experiments.

### Zymography

**Fig. 1:**
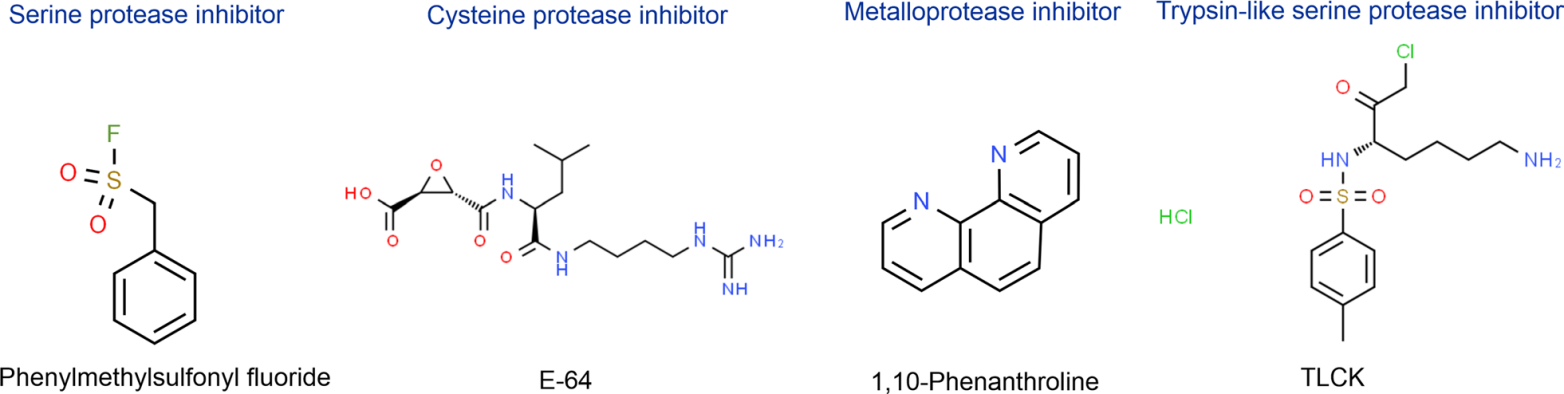
Main classes and general structures of protease inhibitors used (source structures ChemSpider)

The electrophoretic profiles of secreted proteases were evaluated using sodium dodecyl sulfate–polyacrylamide gels containing 0.2% gelatin, and electrophoresis was performed at 30 mA. Following electrophoresis, the gels were washed twice with 2.5% Triton X-100 for 1 h and once with the developing buffer to remove SDS and incubated overnight at 37 °C with the developing buffer to activate the proteases. Additionally, samples were processed and treated with the previously described protease inhibitors (Fig. 1). Phenanthroline, a reversible inhibitor, was also added to the developing buffer. The gels were stained using Coomassie blue staining solution. The proteolytic activities were detected, captured and analysed using the Gel Doc XR + with Image Lab^™^ Software (Bio-rad Laboratories Inc., Vienna, Austria). To confirm the results obtained by zymography, light microscopy was used to assess the progression of trophozoites and the effect of the amoebae (1BU, SIN20, 1BUH3x and SIN20H3x) on the HCEC monolayers.

### *Acanthamoeba* MBP gene expression during co-culture with corneal epithelial cells

The total RNA of *Acanthamoeba* 1BUH3x was extracted using the RNeasy MiniKit (Qiagen, Germany) according to the manufacturer’s instructions and treated with Turbo^™^ DNase (Invitrogen, USA) to remove DNA contamination. The quality and purity of RNA were checked using a Nanodrop ND-1000 spectrophotometer (NanoDrop Technologies, Inc., Wilmington, DE, USA). Reference genes and their efficiency calculations were used as described [38]; their sequences are listed in Additional file 1: Table S1. The MBP primer sequences were obtained from the literature [36]; the primers were ordered at Eurofins (Wiener Neudorf, Austria), and their specificity was tested using a conventional PCR and 1% agarose gel electrophoresis. To calculate the efficiency of MBP primers, RT-qPCR was used to generate a standard curve using a tenfold serial dilution of RNA. The primer efficiency was calculated using the following formula: *E* = *(10*^*–1/slope*^*—1)* × *100*. The RT-qPCR was performed on a CFX96^™^ Real-Time System (Bio-Rad) using the Luna^®^ Universal One-Step RT-qPCR Kit (New England BioLabs ^®^ Inc., E3005L) and according to the manufacturer’s instructions. The PCR reaction mixture (20 µl) comprised 2 × Luna Universal One-Step Reaction Mix (10 µl), 20 × Luna WarmStart RT Enzyme Mix (1 µl), 10 µM of each primer (0.8 µl) and 50 ng of RNA. The reaction profile included a reverse transcription step for 10 min at 55 °C, an initial denaturation step for 1 min at 95 °C, followed by PCR amplification with 40 cycles of 95 °C for 10 s and 60 °C for 60 s, and a melting curve analysis.

### Cytopathic effect assays and microscopy

The pathogenicity of untreated *Acanthamoeba* and *Acanthamoeba* treated with serine protease inhibitors and their effect on the viability of HCECs were evaluated using the CellTiter 96^®^ AQueous One Solution Reagent as previously described [23]. Phase contrast microscopy was used to observe the effect of this inhibition; a Nikon Eclipse E800 microscope with a Nikon DS-Fi2 camera (Nikon Optoteam, Vienna, Austria) was used for microphotography.

## Results

### Growth rate of *Acanthamoeba* in serum-free medium

The cell integrity of the *Acanthamoeba castellanii* strains 1BU and SIN20 was monitored under various conditions using serum-free corneal epithelial cell medium (CEpiCM), CEpiCM with serum, and peptone yeast extract glucose (PYG) medium, as described in our previous study [23]. In brief, the amoeba count remained constant over 24 h, indicating that they stopped dividing in serum-free CEpiCM. Under all conditions, the amoeba count remained constant for up to 8 h; therefore, this time point was selected for the kinetic experiments and analyses.

### Effect of passaging over human cells on *Acanthamoeba* protease activity

**Fig. 2:**
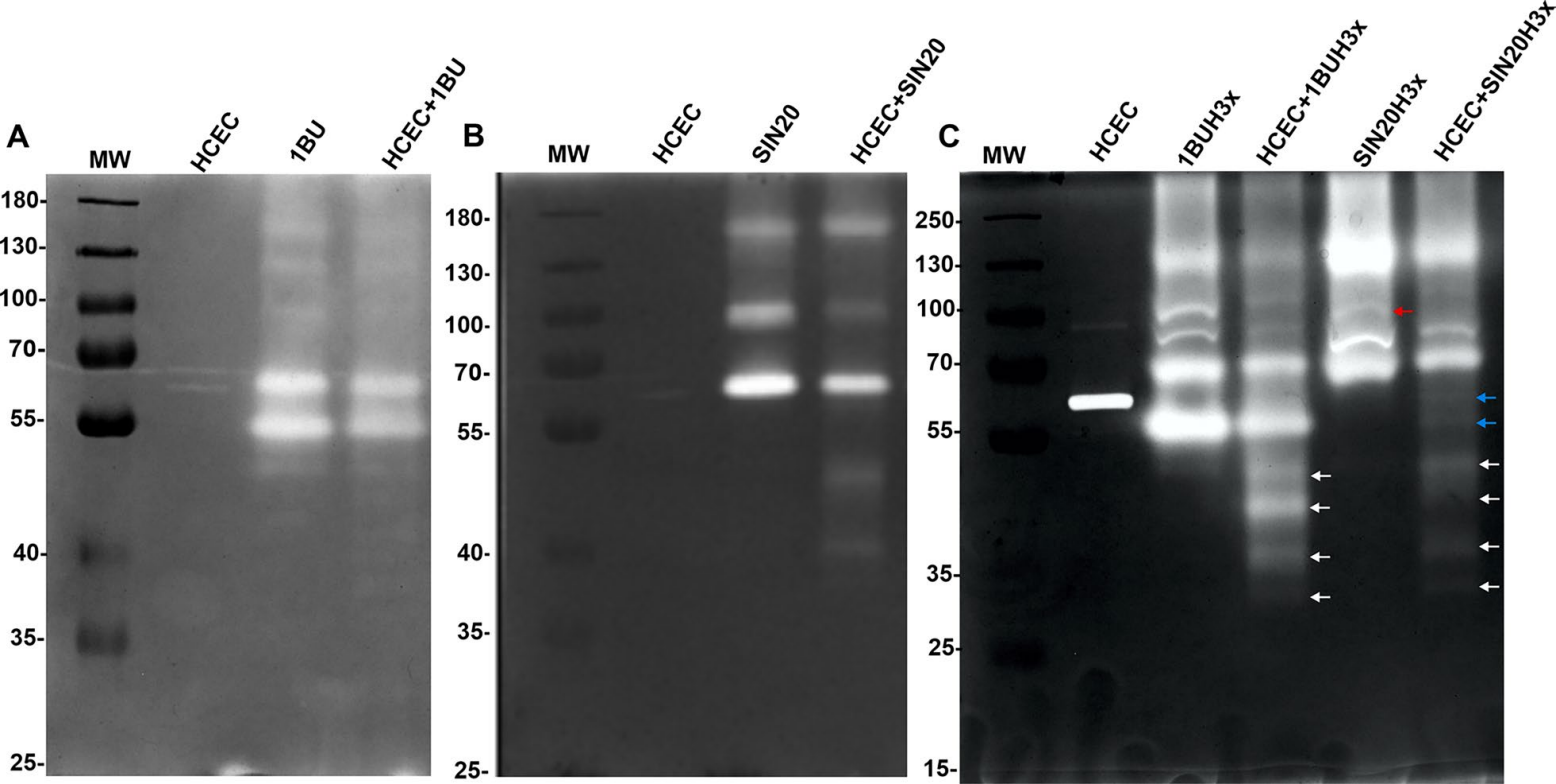
Profiles of extracellular proteases produced by *Acanthamoeba* and HCECs in monoculture and after 1 h of co-culture. The molecular masses are indicated on the left side of each gelatin zymography (10% polyacrylamide). **A** and **B** Proteolytic profiles of *Acanthamoeba* 1BU and SIN20 strains without and during contact with HCECs. **C** Proteolytic profiles of the newly generated subset of strains 1BUH3x and SIN20H3x, without and during contact with HCECs (the red arrow represents the new band displayed by SIN20H3x after reactivation of the attenuated properties, the white arrows the additional proteases displayed after contact with HCECs, and blue arrows proteases released by HCECs)

The zymographic analyses of strains 1BU and SIN20 proteases revealed the presence of discrete bands, and the electrophoretic profiles of the two strains differed (Fig. 2A, B, Additional file 2: Fig. S3). Three passages over cell monolayers resulted in new subsets of the strains—1BUH3x and SIN20H3x, respectively—with enhanced protease expression (Fig. 2C). Moreover, SIN20H3x displayed an additional band at approximately 100 kDa, which was not visible in SIN20 likely because of weak expression (Fig. 2C, red arrow). Furthermore, after 1 h of *Acanthamoeba* co-culture with HCECs, the electrophoretic profiles of the new substrains showed the presence of additional low-molecular-weight (MW) bands compared to the original strains 1BU and SIN20 (Fig. 2C, white arrows), indicating higher protease activity.

### Secretion of additional proteases by *Acanthamoeba* during co-culture with human corneal epithelial cells

The secretion kinetics of extracellular proteases released into serum-free medium by 1BUH3x and SIN20H3x were investigated at 1, 2, 4, 6 and 8 h of co-culture with HCECs. The electrophoretic profiles of both strains in co-culture (compared with their controls, i.e. amoeba monocultures) showed at least four additional protease activity bands corresponding to the MW of approximately 33, 39, 47 and 50 kDa, respectively (Figs. 2C arrows, 3A, B). These bands appeared at 1 h of co-incubation, became prominent at 2 h, indicating active secretion at this time point, and then disappeared after 4 h. Except for an additional band at 55 kDa observed for 1BUH3x, the two strains showed a similar pattern of high-MW protease bands (> 60 kDa).

**Fig. 3:**
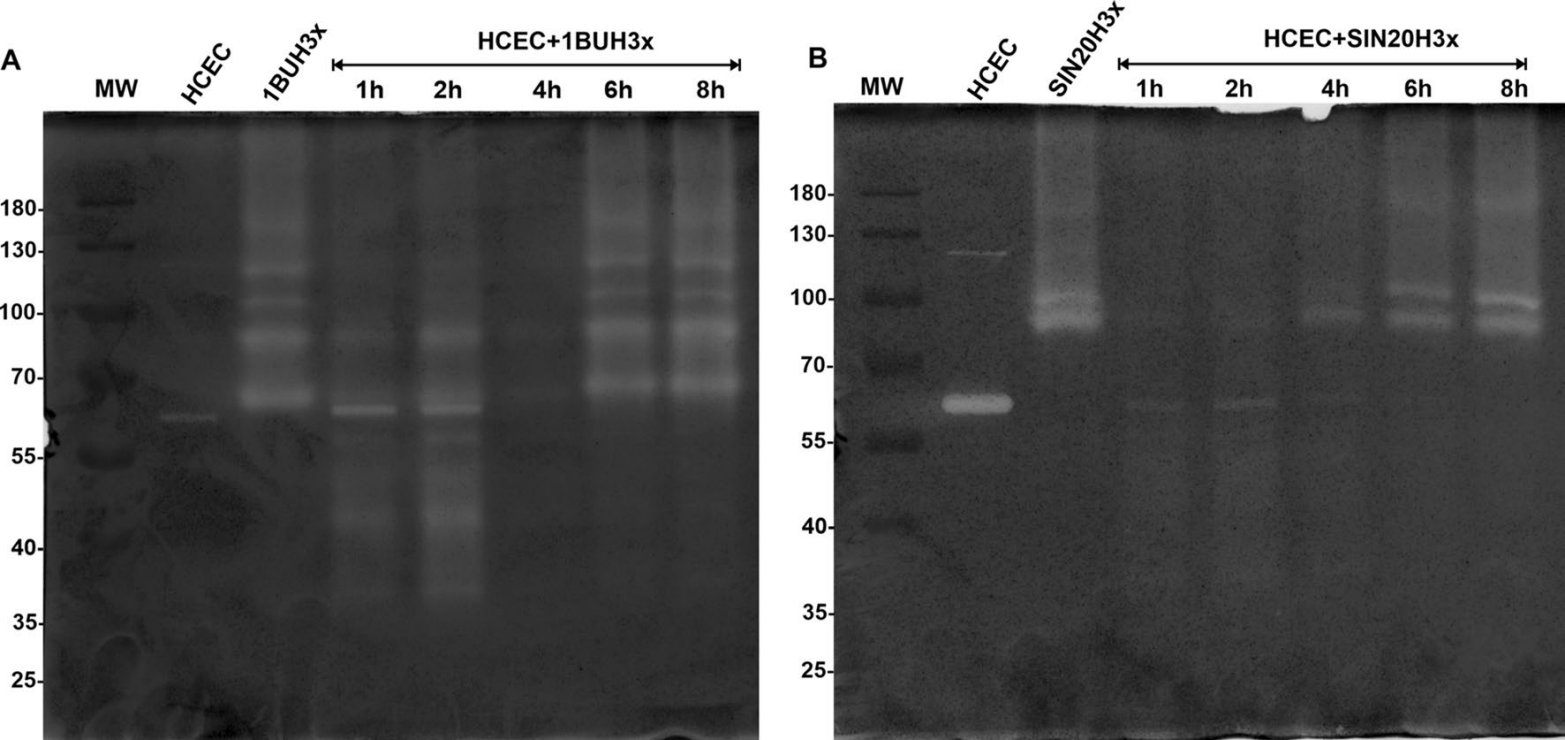
In vitro secretion kinetics of proteases secreted by 1BUH3x (**A**) and SIN20H3x (**B**) after contact with HCECs using 1D in-gel zymography. The patterns of extracellular proteases displayed by HCECs and *Acanthamoeba* in monoculture were used as experimental controls

To exclude the possibility that the apparent increase in protease activity, partly located in low MW, in the serum-free CEpiCM during the first 2 h of co-culture was due to changes in the conditioned medium during co-culture, two experiments were performed. First, serum-free medium from *Acanthamoeba* was collected in a time-dependent manner (1 h, 2 h, 4 h, 6 h and 8 h) and assessed. No change in protease expression was observed during the time course. Second, the pH of the cell-free filtrate was determined; the pH ranged between 7.3 and 7.5 and did not significantly differ between conditioned and unconditioned serum-free CEpiCM.

### Protease inhibitor assays revealed serine proteases as major virulence factors

**Fig. 4:**
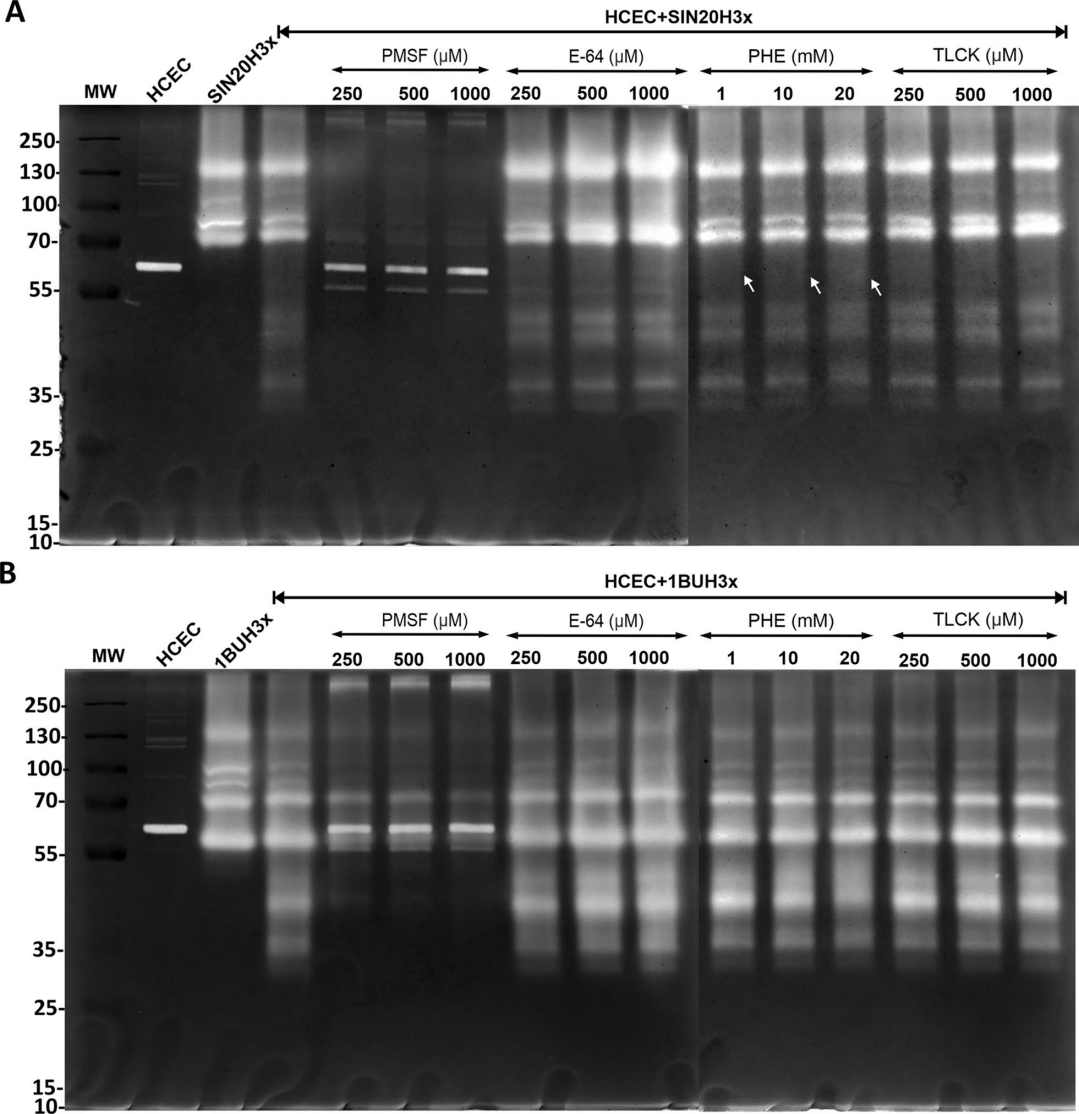
Electrophoretic profiles of *Acanthamoeba* strains SIN20H3x (**A**) and 1BUH3x (**B**) and dose-dependent inhibitory effects of PMSF, E-64, PHE and TLCK. **A** Arrows indicate the absence of two bands after exposure to phenanthroline (PHE)

The mechanistic classes of extracellular proteases secreted by *Acanthamoeba* during their co-culture with HCECs were characterized based on their susceptibility to different protease inhibitors such as PMSF (irreversibly inhibits serine proteases), E-64 (targets cysteine proteases), TLCK (inhibits trypsin-like serine proteases) and phenanthroline (reversible inhibitor of metalloproteases) (Figs. 1 and 4). The susceptibilities of nine protease bands (approximate MW: 33, 39, 47, 50, 55, 70, 80, 100 and 130 kDa) to different protease inhibitors were investigated; the effects were observed to be dose-dependent. Treatment with PMSF strongly affected the banding pattern, even at a low concentration. Additional bands were observed after treatment with PMSF, located approximately at the same MWs as the protease bands produced by HCECs in monoculture. These bands were not observed in the presence of phenanthroline alone (Fig. 4A, white arrow), and together with PMSF (Additional file 2: Fig. S1), indicating that these may be matrix metalloproteinases (MMPs), which are proteases typically secreted by HCECs [24, 25].

### Specificities of proteases secreted by *Acanthamoeba* during contact with human corneal epithelial cells

**Fig. 5:**
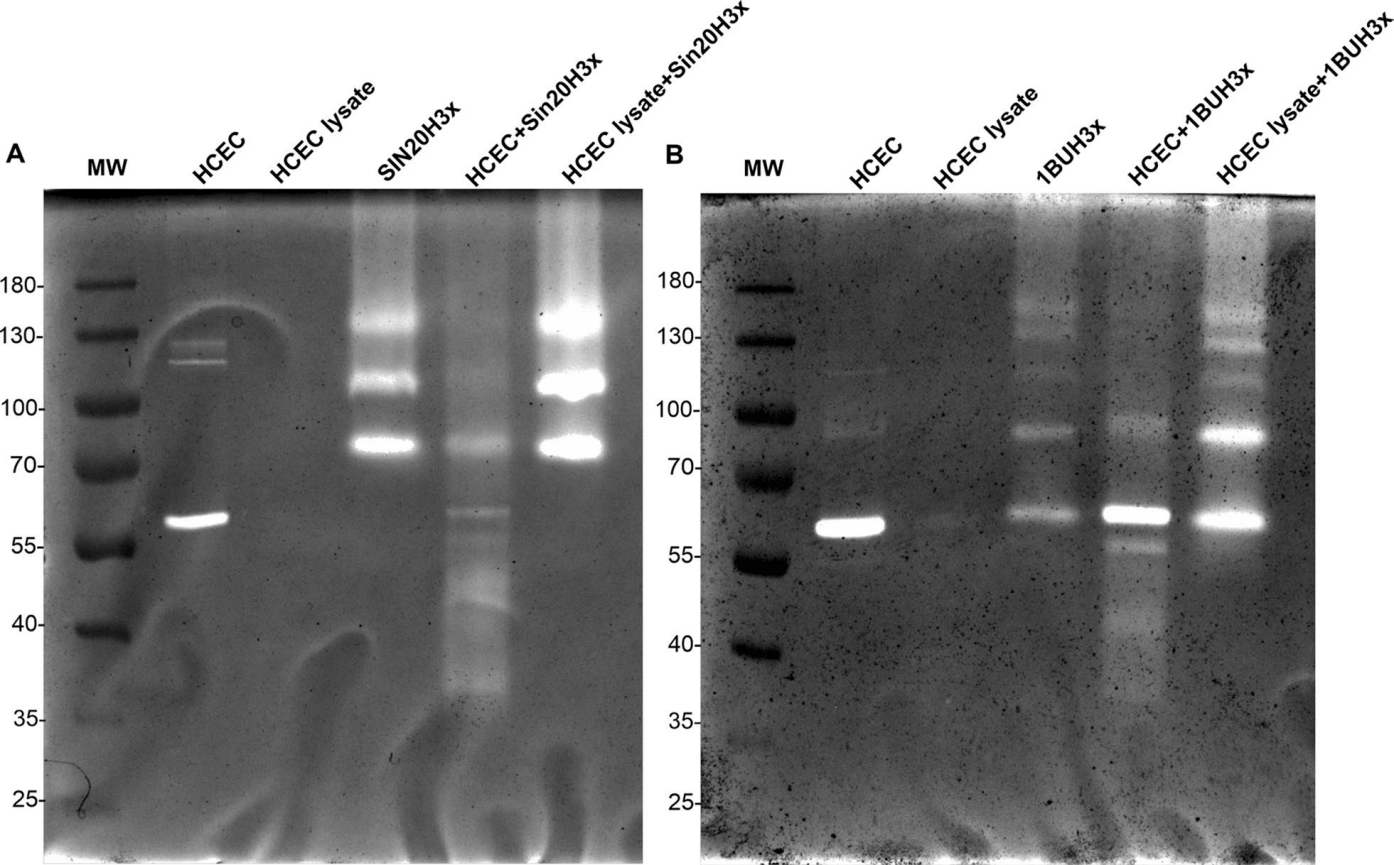
Specificities of proteases secreted by *Acanthamoeba* during contact with HCECs. Proteolytic patterns of *Acanthamoeba* strains SIN20H3x (**A**) and 1BUH3x (**B**) after 1 h of co-culture with HCECs and HCEC lysate

**Fig. 6:**
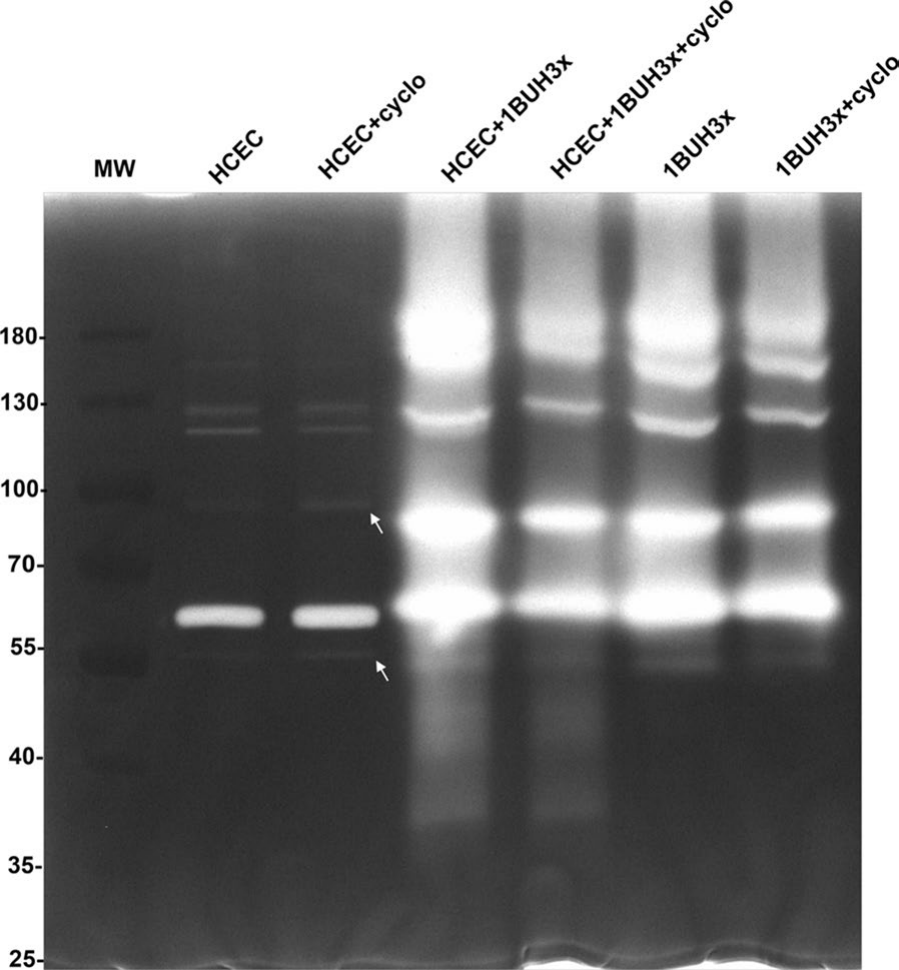
Effect of the protein synthesis inhibitor cycloheximide (100 µM) on the secretion of extracellular proteases produced by HCECs and 1BUH3x in mono- and co-culture. The arrow shows the prominence of a protease band produced by HCECs after 1 h of exposure to cycloheximide

To examine the specificities of additional proteases secreted by *Acanthamoeba*, a control experiment using HCEC lysate (containing cell contents such as proteins, nucleic acids and impurities from the host cells) and another experiment using cycloheximide—a protein synthesis inhibitor—were performed. As shown in Fig. 5, the contact with HCEC lysate did not promote the release of additional proteases by *Acanthamoeba*; however, the expression of additional proteases was triggered and appeared more pronounced when *Acanthamoeba* was cultured with living HCECs. Remarkably, treatment with 100 µM cycloheximide had no noticeable effect after 1 h of co-culture; however, it seemed to be effective with HCECs, which exhibited moderate protease bands at 1 h after exposure to this compound (Fig. 6, white arrows).

### Protease activity did not correlate to MBP gene expression

**Fig. 7:**
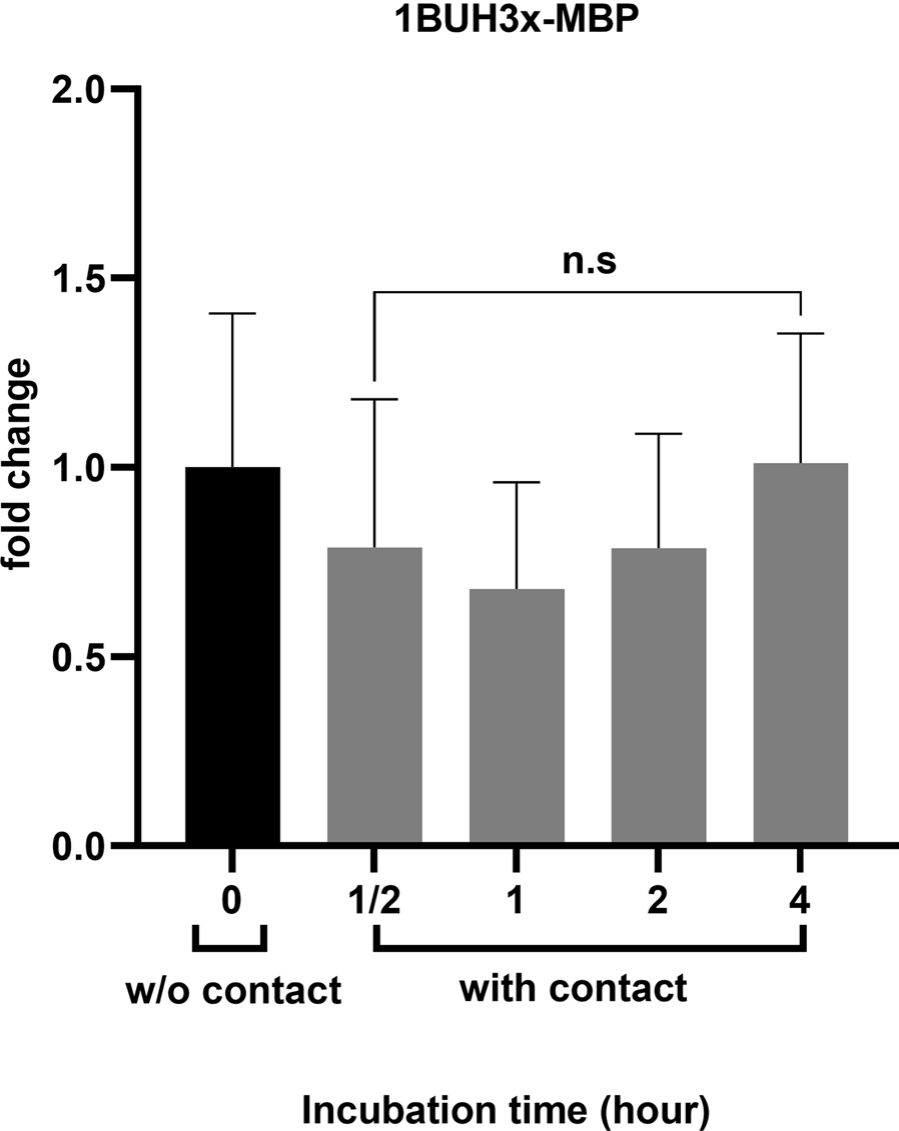
Expression of the *MBP* genes in *Acanthamoeba* 1BUH3x before (black bar) and after (grey bars) contact with HCECs. The y-axis indicates the fold increase in mRNA levels compared to the control (without contact). The error bars show the standard deviation of the mean (SEM). Values were obtained from at least three biological replicates in duplicate. ns: non-significant according to statistical analysis (Student's *t*-test)

To determine whether the appearance of new protease bands secreted by *Acanthamoeba* immediately after contact with HCECs is associated with the expression of *MBP*, the expression level of *MBP* was compared under various conditions (Fig. 7). The *Acanthamoeba* 1BU strain was isolated 25 years ago, and the reactivation of its attenuated properties by passage over human cell monolayers with the emergence of a new substrain (1BUH3x), which is distinct from the non-reactivated strain, has been previously reported [16]; therefore, this strain was used in this experiment. Changes in *MBP* gene expression from monocultured *Acanthamoeba* and from *Acanthamoeba* co-cultured with HCECs are shown in Fig. 7. Remarkably, no significant changes were observed in *MBP* gene expression after 30 min, 1 h, 2 h and 4 h of co-culture compared with the control. The gene was highly expressed before and after contact with HCECs, with cycle threshold (Ct) values between 16 and 17. Notably, the expression decreased slightly after 1 h of co-culture and then returned to its initial levels.

### Inhibition of serine proteases reduced the cytotoxicity of *Acanthamoeba*

**Fig. 8:**
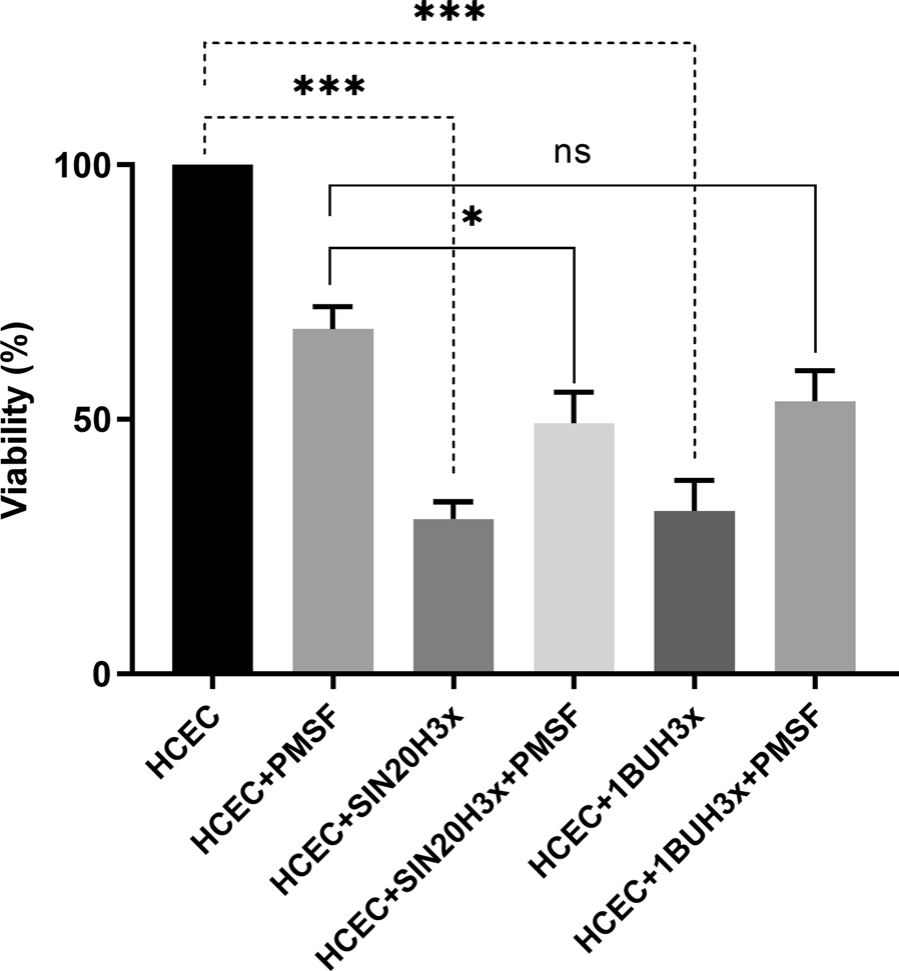
Viability of untreated and PMSF-treated (250 µM) HCECs co-cultured with *Acanthamoeba* 1BUH3x and SIN20H3x. The time of exposure to the amoeba or/and the inhibitor was 8 h. Values represent the mean of four independent experiments, each in triplicate. Statistical analysis was performed through two-way ANOVA with Dunnett’s multiple comparisons test (*P < 0.01, **P < 0.001, ***P < 0.0001 and ns: non-significant)

**Fig. 9:**
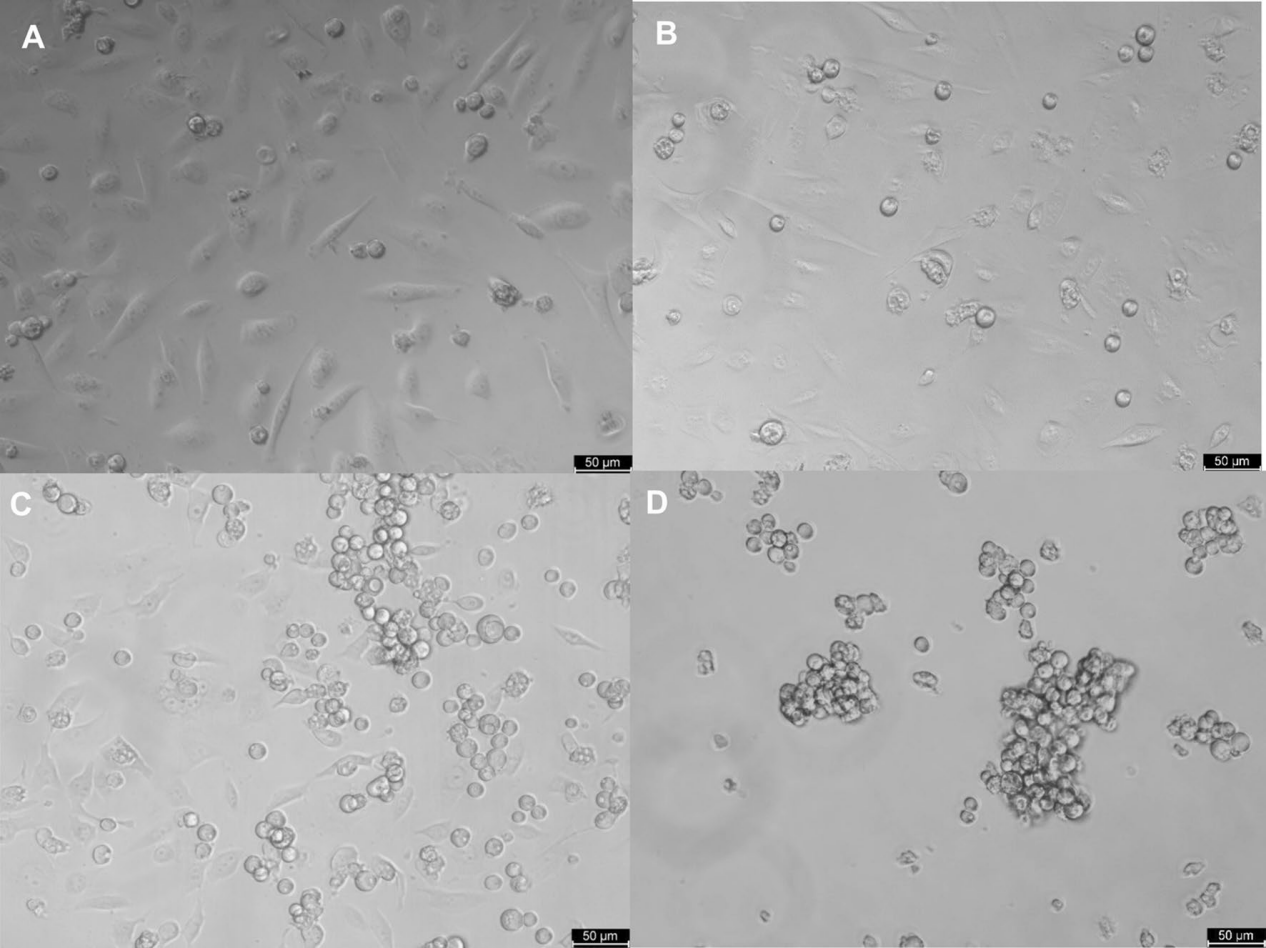
Phase contrast microscopy of *Acanthamoeba* 1BUH3x in monoculture and co-culture with HCECs in the presence or absence of PMSF. **A** HCECs in monoculture; **B** 1BUH3x attached to the flask and bound to HCECs; **C** *Acanthamoeba* (aggregate, upper part) in co-culture with HCECs, forming clusters and not attaching and binding on the flask and the mammalian cells because of the presence of the inhibitor; **D** aggregation of amoebae in monoculture due to the presence of PMSF. Scale bar: 50 µm

To investigate whether serine proteases play an important role in the induction of host cell death by *Acanthamoeba*, HCECs were treated with PMSF—a cell-permeable, irreversible inhibitor of serine proteases—before they were inoculated with the strains 1BUH3x and SIN20H3x. At 6 h post inoculation, the viability of HCECs was evaluated using the CellTiter 96^®^ AQueous One Solution Reagent, which contains a tetrazolium salt, which is reduced to formazan by viable mammalian cells and not by *Acanthamoeba* [23]. The viability of untreated and treated HCECs co-cultured with *Acanthamoeba* was assessed by measuring the quantity of formazan produced by the mammalian host cells (Fig. 8). *Acanthamoeba* had a lower cytotoxic effect on PMSF-treated HCECs than on untreated HCECs. Moreover, microscopic studies revealed that the amoebae were unable to bind to the host cells in the PMSF-treated group, which reduced their cytopathic effect (Fig. 9). This finding suggests that the serine proteases of *Acanthamoeba* play a role not only in cytopathogenicity but also in adhesion to host cells.

## Discussion

In this study, we examined the lytic factors produced by *A. castellanii* during the early phase of interaction with human corneal epithelial cells (HCECs) by using 1D in-gel zymography. We observed the emergence of four protease bands ranging from 33 to 50 kDa in MW, secreted by *A. castellanii* during the first 2 h of co-incubation with HCECs. The sequential passaging of *Acanthamoeba* strains over human cell monolayers significantly enhanced the activity of proteases, including those newly displayed, demonstrating the reactivation of attenuated properties and their involvement in *Acanthamoeba* virulence. The novel protease bands were not observed after 4, 6 and 8 h of co-incubation. All protease bands, including the new bands, were susceptible to serine protease inhibitors. The presence of PMSF prevented the binding of *Acanthamoeba* to HCECs as well as the induction of cytolysis. Interestingly, the expression of *Acanthamoeba* mannose-binding protein did not differ between amoebae in monoculture and those in co-culture.

Proteases play a crucial role for *Acanthamoeba* survival in the human host, enabling the amoebae to invade human tissue and to degrade host cells and extracellular matrix components [2, 9, 26]. Among all extracellular proteases produced by *Acanthamoeba*, a 133-kDa and a 40-kDa serine protease, termed mannose-induced protein (MIP-133) and plasminogen activator (PA), respectively, have been identified as essential contributors to *Acanthamoeba* pathogenesis [21, 27]. In the current study, we showed that *Acanthamoeba* in monoculture secretes various proteases, all high MW, ranging between approximately 55 and 130 kDa. It is likely that the prominent ~ 130 kDa protease corresponds to MIP-133, which possesses cytolytic properties and is evidently associated with cytopathogenicity. Furthermore, the proteolytic activity increased over time (up to 8 h), indicating an active secretion of extracellular proteases (Additional file 2: Fig. S2). The pattern of secreted proteases by 1BUH3x and SIN20H3x in monoculture was identical over time, and no change was observed (Additional file 2: Fig. S2).

Strain 1BU, when isolated > 25 years ago from a patient with AK, exhibited a high cytopathic effect on HEp-2 cells [28]. This cytopathic effect was found to decline during long-term axenic culture, but was strongly reactivated when the strain was passaged over a HEp-2 cell monolayer [16], which is consistent with the present findings of pronounced extracellular proteolytic activity of this isolate. A similar observation was made with the recent isolate, SIN20, as the new substrain SIN20H3x, obtained by three passages over human cells, displayed a new protease band at approximately 100 kDa (Fig. 2C, red arrow). This newly emerged band may represent a protease described previously, which corresponds to the type of protease expressed only by pathogenic isolates and which appears after reactivation of attenuated properties [16, 29].

In the present study, the strains SIN20H3x and 1BUH3x showed four and five major bands, respectively, before contact with HCECs. This is consistent with recent reports in which the proteolytic activities of several *Acanthamoeba* T4 isolates from various origins were examined, and it was found that each isolate displayed different patterns and up to five prominent bands on zymogram gels, which were classified as belonging to the serine protease group [30, 31]. During the first 2 h after contact with HCECs, both strains displayed new bands of proteolytic activity in addition to those previously expressed, including those corresponding to matrix metalloproteinases (MMPs) secreted by HCECs, which were clearly visible after co-culturing with SIN20H3x (Fig. 2C, blue arrow) and in the presence of PMSF (Fig. 4A, B). These MMPs (around 55 kDa) produced by the HCECs after co-culture may correspond to the MMPs reported by Alizadeh et al., which are produced and activated upon contact with *Acanthamoeba*, leading to the degradation of the extracellular matrix [25]*.* A similar banding pattern was also observed when the HCECs were exposed to cycloheximide (Fig. 6, arrows), suggesting that these MMPs appear during cytolysis. Moreover, it seems that most of the proteases exhibited by *Acanthamoeba* were already synthesized, as observed in a previous study [32]. Thus, cycloheximide was ineffective on *Acanthamoeba* (Fig. 6). Previous studies using zymography assays evaluated the protease profiles of *Acanthamoeba* after 24 h interaction with Madin-Darby canine kidney (MDCK) cells and found that the patterns were unchanged [13, 29]. This distinct finding could be related to the long incubation period in which events, occurring during the early phase of interaction between the amoebae and the host cells, may remain unnoticed. Thus, the focus of this study was on the secretion kinetics of *Acanthamoeba* proteases during the first 8 h of contact.

Of the additional proteases secreted by *Acanthamoeba* after contact with HCECs, two proteases with a MW of approximately 33 kDa and 39 kDa may correspond to the 33-kDa subtilase and 40-kDa PA described by Kim et al. and Mitra et al., respectively, which have cytopathic effects and are crucial in host tissue invasion by *Acanthamoeba* [27, 33]. To our knowledge, this is the first study to describe the simultaneous production of these proteases by *Acanthamoeba*. Two other proteases (MW approximately 47 and 50 kDa), similar to those indicated above, may also play an important role in *Acanthamoeba* pathogenicity. However, further investigation (i.e. protein identification by mass spectrometry) is necessary to confirm this hypothesis.

Most of the additional protease activity occurred within the first 2 h of co-culture with human corneal epithelial cells, corresponding to an event occurring immediately after contact with the host cells. This finding indicates that the binding of *Acanthamoeba* to the host cells triggers the secretion of new proteins capable of inducing host cell death. Because several studies have suggested that MBP, a glycoprotein on the surface of *Acanthamoeba* trophozoites, plays a key role in host cell adhesion [14, 34, 35], we quantified the mRNA expression of the *MBP* gene before and after contact with HCECs. *MBP* was highly expressed in the new substrain of the *Acanthamoeba* 1BU strain, which is consistent with the findings of Ng et al., who reported that virulent strains showed a higher level of *MBP* gene expression than non-virulent strains [36]. However, no difference in expression was observed before and after contact with HCECs and up to 4 h of interaction in the present study (Fig. 7).

The kinetics of proteases are influenced by the parasite, host response and environmental conditions [9, 37]. The transient nature of the observed proteolytic activities is particularly interesting. In our previous study, we showed that the *Acanthamoeba* strains 1BU and SIN20 were able to reduce cell viability by 32.31% and 21.40% within 4 h and 6.36% and 4.6% within 8 h of co-culture with HCECs, respectively [23]. Thus, the comparably short time of activity of the novel proteases found in the current study may be attributed to the reduced viability of the HCECs during co-culture with amoebae, again leading to a decrease in protease secretion by the amoebae. In this study, we only investigated two different strains of *A. castellanii*, both genotype T4; but we assume that our observations may be extended to other pathogenic *Acanthamoeba* strains, since all clinical isolates investigated so far and also several environmental isolates have been shown to exhibit cytolytic activities [9, 13, 29]. However, further investigation will be needed to confirm this hypothesis and to elucidate the kinetics of these proteases.

## Conclusions

In summary, our findings revealed that, in addition to mannose-induced cytopathic protein MIP-133 and plasminogen activator, other serine proteases may be involved in *Acanthamoeba* pathogenesis and demonstrated the production and activation of metalloproteinases in host cells after contact with *Acanthamoeba*, which may promote host tissue invasion. Notably, the prominent proteolytic activity during the first hours of cell–cell interaction was not correlated with *MBP* gene expression, although this gene was highly expressed by *Acanthamoeba* before and after contact with HCECs.

### Supplementary Information

**Additional file 1: Table S1.** List of primers used in this study.

**Additional file 2 Figure 1.** Effect of PMSF alone and PMSF associated with phenanthroline (PHE) on the protease secreted by *Acanthamoeba *during co-culture with HCECs. MW represents the molecular weight. The first column represents the protease secreted by *Acanthamoeba* 1BUH3x in monoculture. The second column represents proteases secreted by 1BUH3x in contact with HCECs without inhibitors, the third in the presence of PMSF (1 mM) and the fourth in the presence of PMSF (1 mM) associated with phenanthroline (PHE, 20 mM). **Figure 2.** Patterns of extracellular proteases produced by *Acanthamoeba *1BUH3x (A) and SIN20H3x (B) in monoculture. The inoculums (1 × 10^6^ amoebae) were loaded into T25 flasks for cell culture containing a serum-free CEpiCM (total volume: 6 ml) and incubated for various periods (1 h, 2 h, 4 h, 6 h and 8 h). At each time point, the culture medium was collected and filtered. A volume of 10 µl for each sample was collected and used to evaluate the proteolytic activity over time.** Figure 3.** In vitro secretion kinetics of proteases secreted by 1BU (A) and SIN20 (B) after contact with HCECs using 1D in-gel zymography. The patterns of extracellular proteases displayed by HCECs and *Acanthamoeba*, in monoculture, were used as experimental controls.

## Data Availability

All data generated or analyzed in this study are included within the article and its additional file.
