# Supplementary material for: Characterization of novel extracellular proteases produced by Acanthamoeba castellanii after contact with human corneal epithelial cells and their relevance to pathogenesis
Source: Parasit Vectors. 2024 May 29;17:242. doi: 10.1186/s13071-024-06304-7 (PMC11137893; doi:10.1186/s13071-024-06304-7)
Supplement: Supplementary file 1 — Additional file 1: Table S1. List of primers used in this study. [file 13071_2024_6304_MOESM1_ESM.docx]

Table S1: List of primers used in this study

| Target Genes | Target | Direction | Primer Sequence (5´-3´) | Amplicon (bp) | Source |
| --- | --- | --- | --- | --- | --- |
| 18S-rRNA-gene | 18SQ | Forward | CCCAGATCGTTTACCGTGAA | 180 | (38) |
|  |  | Reverse | TAAATATTAATGCCCCCAACTATCC |  |  |
| Hypoxanthine-guanine  phosphoribosyltransferase | HPRT | Forward | GGAGCGGATCGTTCTCTG | 201 |  |
|  |  | Reverse | ATCTTGGCGTCGACGTGC |  |  |
| Mannose-binding protein | MBP | Forward | AGGGCGAGACCTACGATAGC | 165 | (36) |
|  |  | Reverse | CCTCGTAGACGAAGGTGAGG |  |  |
