# Supplementary material for: Characterization of novel extracellular proteases produced by Acanthamoeba castellanii after contact with human corneal epithelial cells and their relevance to pathogenesis
Source: Parasit Vectors. 2024 May 29;17:242. doi: 10.1186/s13071-024-06304-7 (PMC11137893; doi:10.1186/s13071-024-06304-7)
Supplement: Supplementary file 2 — Additional file 2 Figure 1. Effect of PMSF alone and PMSF associated with phenanthroline (PHE) on the protease secreted by Acanthamoeba during co-culture with HCECs. MW represents the molecular weight. The first column represents the protease secreted by Acanthamoeba 1BUH3x in monoculture. The second column represents proteases secreted by 1BUH3x in contact with HCECs without inhibitors, the third in the presence of PMSF (1 mM) and the fourth in the presence of PMSF (1 mM) associated with phenanthroline (PHE, 20 mM). Figure 2. Patterns of extracellular proteases produced by Acanthamoeba 1BUH3x (A) and SIN20H3x (B) in monoculture. The inoculums (1 × 10^6^ amoebae) were loaded into T25 flasks for cell culture containing a serum-free CEpiCM (total volume: 6 ml) and incubated for various periods (1 h, 2 h, 4 h, 6 h and 8 h). At each time point, the culture medium was collected and filtered. A volume of 10 µl for each sample was collected and used to evaluate the proteolytic activity over time. Figure 3. In vitro secretion kinetics of proteases secreted by 1BU (A) and SIN20 (B) after contact with HCECs using 1D in-gel zymography. The patterns of extracellular proteases displayed by HCECs and Acanthamoeba, in monoculture, were used as experimental controls. [file 13071_2024_6304_MOESM2_ESM.docx]

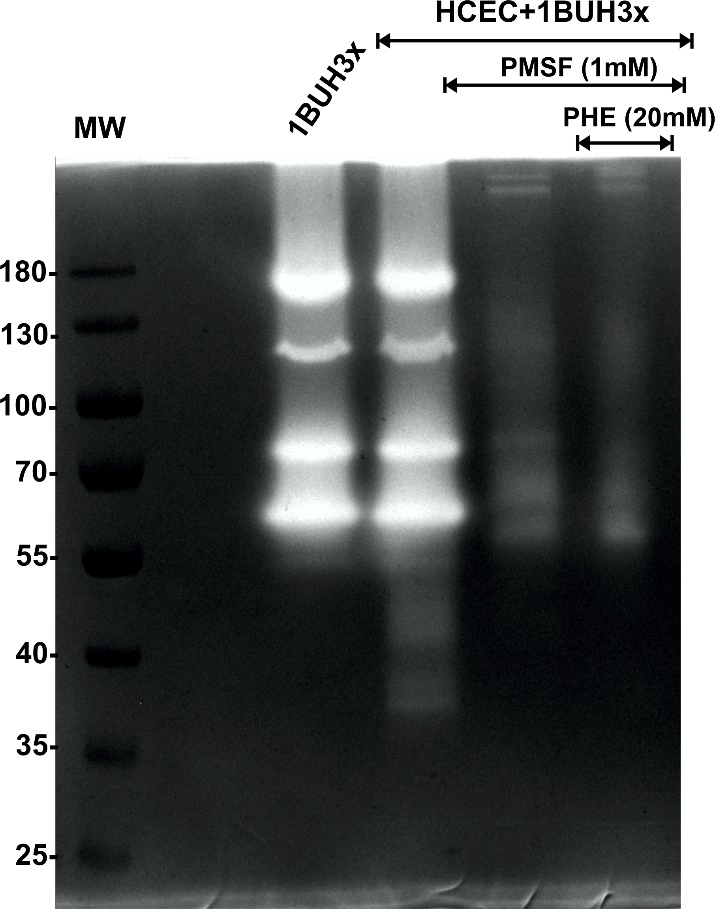


**Additional file 2: Figure 1**. Effect of PMSF alone, and PMSF associated with phenanthroline (PHE) on the protease secreted by *Acanthamoeba* during co-culture with HCECs. MW represents the molecular weight. The first column represents the protease secreted by *Acanthamoeba* 1BUH3x in monoculture. The second column represents proteases secreted by 1BUH3x in contact with HCECs without inhibitors, the third one in the presence of PMSF (1 mM), and the fourth in the presence of PMSF (1 mM) associated with phenanthroline (PHE, 20 mM).


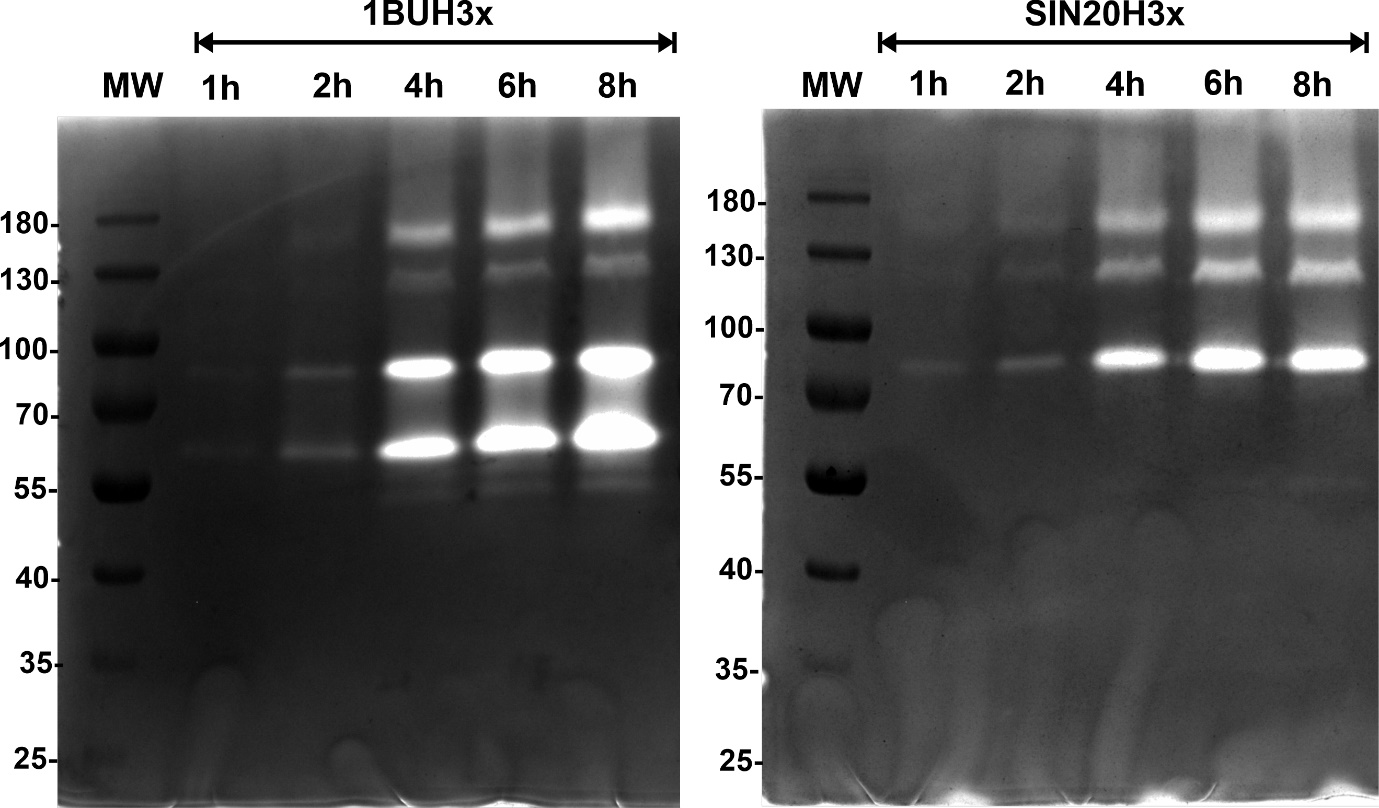


**Additional file 2: Figure 2**. Patterns of extracellular proteases produced by *Acanthamoeba* 1BUH3x (A) and SIN20H3x (B) in monoculture. The inoculums (1x10^6^ amoebae) were loaded into T25 flasks for cell culture, containing a serum-free CEpiCM (total volume: 6 mL) and incubated for various periods (1 h, 2 h, 4 h, 6 h and 8 h). At each time point, the culture medium was collected and filtered. A volume of 10 µl for each sample was collected and used, to evaluate the proteolytic activity over time.


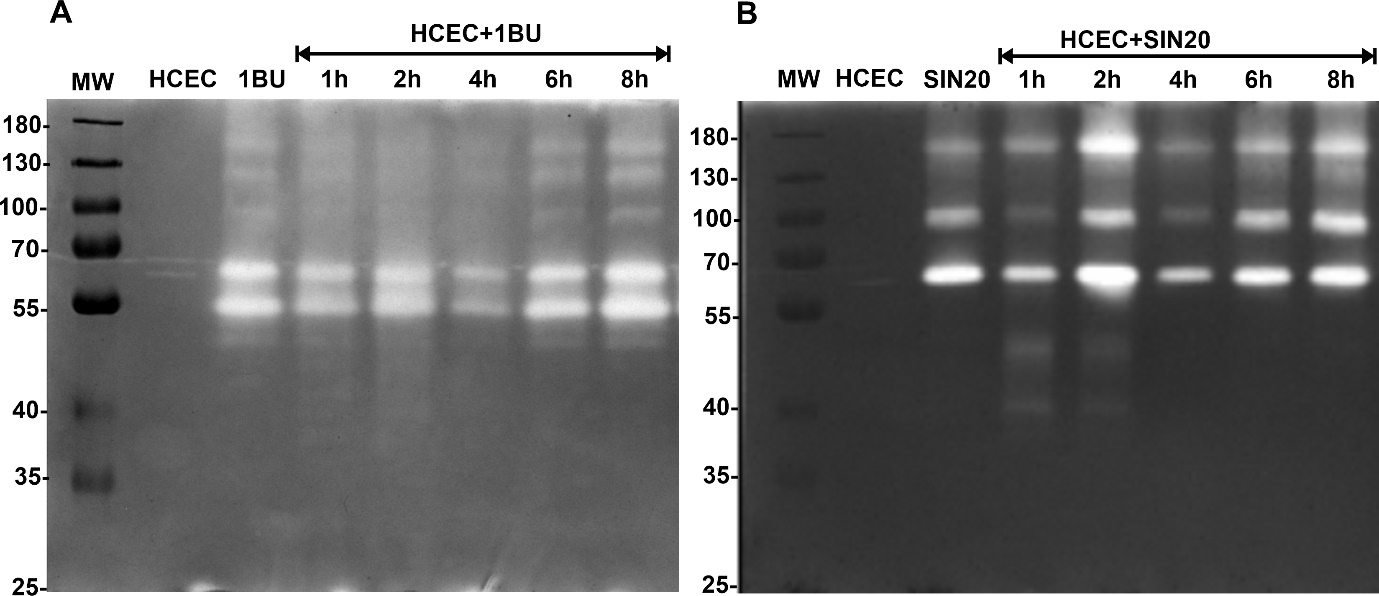


**Additional file 2: Figure 3**. *In vitro* secretion kinetics of proteases secreted by 1BU (A) and SIN20 (B) after contact with HCECs, using 1D in-gel zymography. The patterns of extracellular proteases displayed by HCECs and *Acanthamoeba*, in monoculture, were used as experimental controls.
